# Pacemaker Implantation in a Patient With Isolated Persistent Left Superior Vena Cava: A Novel Approach

**DOI:** 10.1002/ccr3.70031

**Published:** 2025-01-12

**Authors:** Shayan Shahi, Soroush Nematollahi, Ali Vasheghani‐Farahani

**Affiliations:** ^1^ Tehran Heart Center Cardiovascular Diseases Research Institute, Tehran University of Medical Sciences Tehran Iran; ^2^ Cardiac Primary Prevention Research Center Cardiovascular Diseases Research Institute, Tehran University of Medical Sciences Tehran Iran

**Keywords:** electrophysiology, pacemaker, persistent left superior vena cava, venous anomalies

## Abstract

Implanting pacemakers in patients with isolated persistent left superior vena cava (PLSVC) present unique challenges. Recognizing venous anomalies and adapting lead placement techniques are crucial for successful outcomes and stable pacemaker function.

## 1 Introduction

Thoracic systemic venous anomalies, a group of vascular disorders, are usually symptom‐free and are often discovered accidentally during medical procedures or surgeries unless other congenital heart conditions accompany them [1]. The most common anomaly among these is the persistent left superior vena cava (PLSVC), which affects 0.5%–2% of the general population. In most cases (90%), the PLSVC drains blood into the right atrium (RA) [1, 2]. Typically, the PLSVC coexists with the normal right‐sided superior vena cava (SVC) system, but in 10%–20% of cases, the right‐sided SVC is absent, leaving only the isolated PLSVC [1].

The following case involves a patient with an isolated PLSVC draining into the RA, along with an absent right SVC. This patient presented with an irreversible high‐grade atrioventricular block, which made the implantation of a dual‐chamber pacemaker challenging. Electrophysiologists typically implant a permanent pacemaker (PPM) via the left brachiocephalic vein, draining into the right SVC, RA, and right ventricle (RV). The absence of the right SVC in a patient with PLSVC presents a challenge when implanting a PPM in these cases.

## 2 Case History and Examination

A 67‐year‐old woman presented to our emergency department (ED) complaining of dizziness that began 3 days ago. She denied any chest pain or difficulty breathing and had no history of fainting. Her medical history included Type II diabetes mellitus (T2DM) and hypertension (HTN). Her current medications were valsartan 80 mg twice a day, hydrochlorothiazide 25 mg daily, and metformin 500 mg three times a day.

**FIGURE 1:**
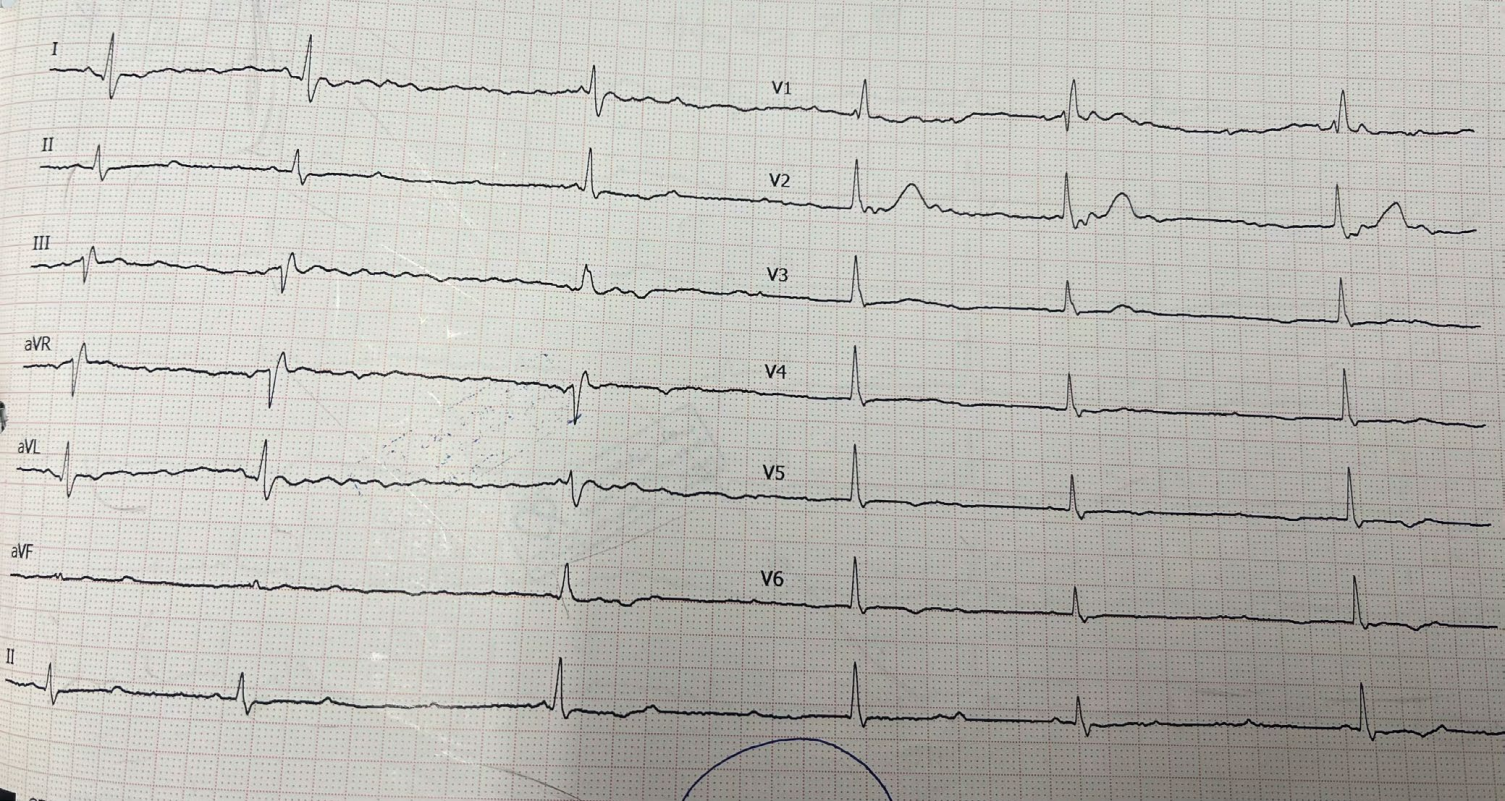
An electrocardiogram of the patient upon her arrival at the emergency department revealed a high‐grade atrioventricular block.

Upon her arrival at the emergency department, the ECG showed a high‐grade atrioventricular block (Figure 1). Her initial lab results, including electrolytes and troponin levels, were normal. The bedside echocardiography revealed normal left and right ventricular function and no significant valvular heart disease. Due to an unstable and wide QRS escape rhythm, we admitted the patient to our ED. Per our routine practice, a temporary pacemaker was inserted via the right femoral vein, and the patient was scheduled for permanent pacemaker implantation the following day.

**FIGURE 2:**
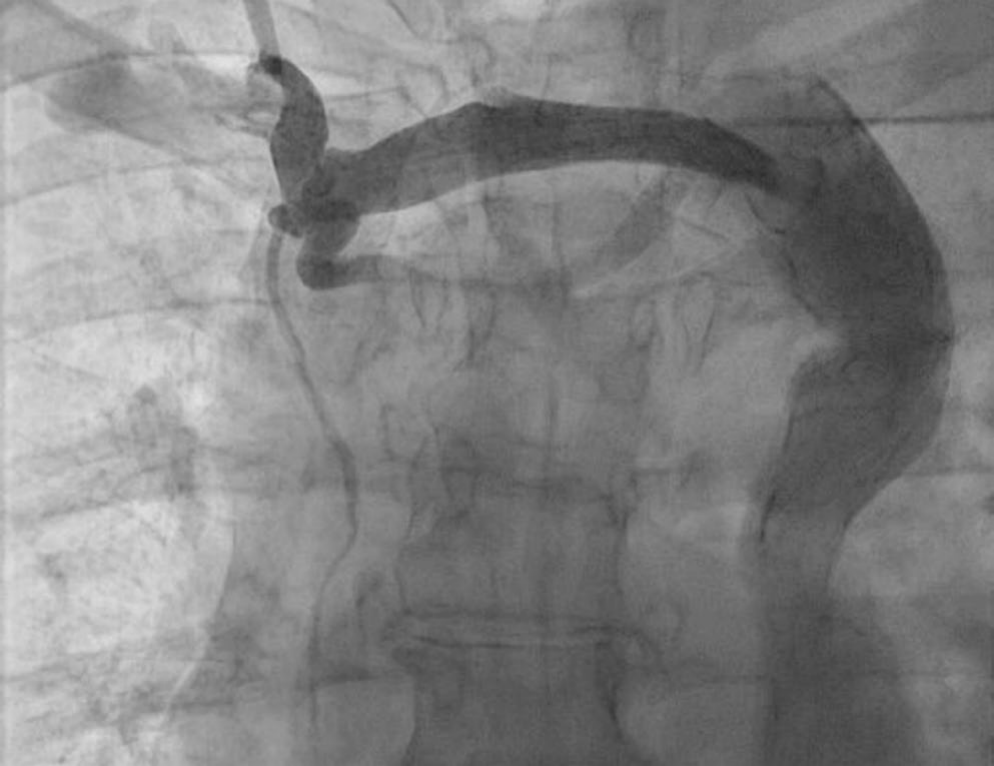
Thoracic venography showed atretic right superior vena cava and left superior vena cava draining into the right atrium.

**FIGURE 3:**
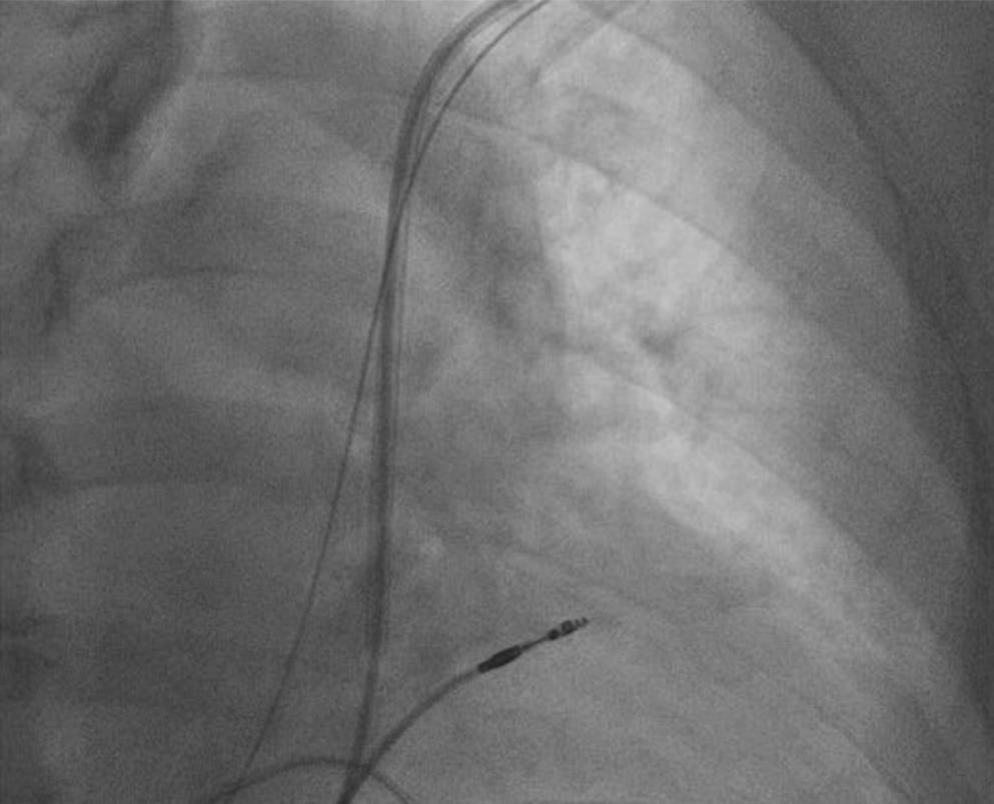
The L‐shaped stylet helped to advance the right ventricular lead.

**FIGURE 4:**
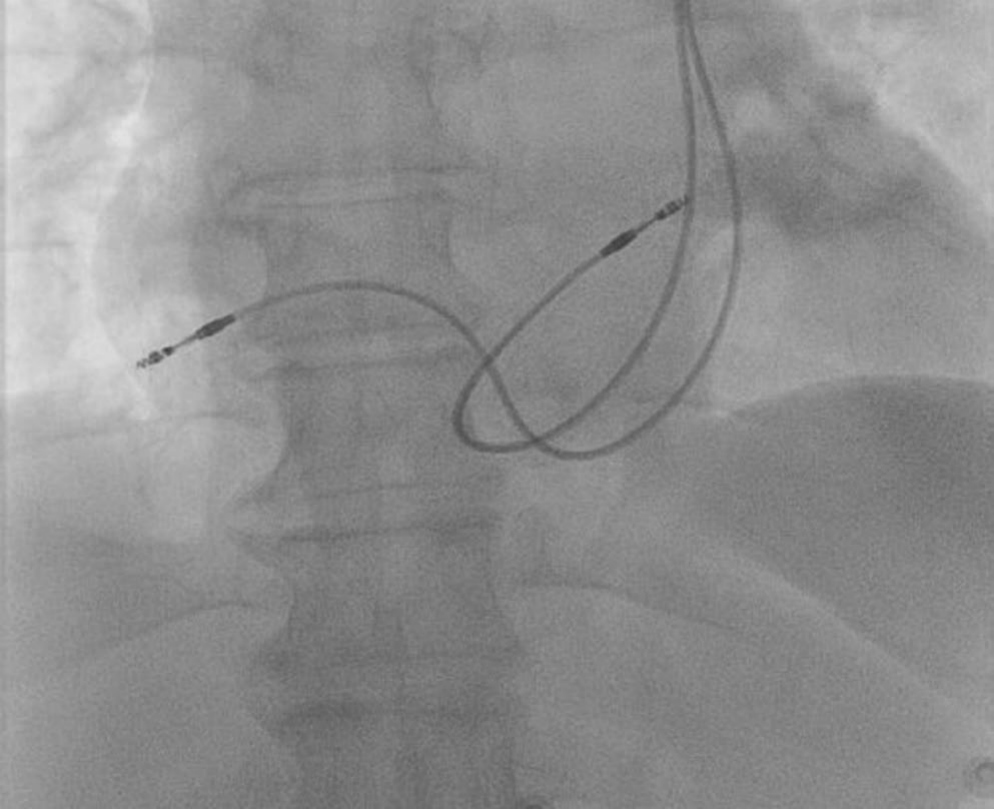
The right atrium and right ventricle are well positioned in the right atrium (RA) appendage and right ventricle outflow tract, respectively.

The next day, the patient had a fever with a body temperature of 38.3°C. As a precaution, we started her on antibiotics, postponed the pacemaker implantation, and consulted an infectious disease specialist. After 4 days of antibiotic treatment, her fever went down, and her body temperature returned to 36.7°C. At this point, we went ahead with the pacemaker implantation. We accessed the left subclavian vein and inserted the sheath during the procedure. However, we faced difficulty guiding the wire into the RA. To get a better view of the anatomy, we performed venography (Figure 2). After further examination, we found that the patient had a blocked right SVC. All the venous blood flow to the RA was through the left superior vena cava (LSVC). This unusual condition made implanting the pacemaker leads, especially the RV lead, challenging. Despite this, we proceeded with the pacemaker implantation via the LSVC. We started by choosing a 58‐cm passive fixation lead and carefully threading the wire through the left SVC and coronary sinus (CS), guiding it around the left side of the spine until it reached the RA. We modified the end of the stylet to have a sharp, L‐shaped curve (see Figure 3) to make it easier to insert into the RV. This allowed us to guide the lead into the RV using the L‐shaped stylet. We created a large loop in the RA near the free wall to provide additional support. As we encountered difficulties securing the passive fixation lead in the RV, we decided to use an active fixation lead instead. The ventricular lead was then positioned at the right ventricular outflow tract (RVOT) by rotating it clockwise and slightly withdrawing it toward the tricuspid annulus. The RA and passive fixation lead were placed in the right atrial appendage using the second sheath. The final position of the double‐pacing leads was assessed using fluoroscopic projections (Figure 4), and pacing parameters were obtained at the end of the procedure.

## 3 Methods

Differential diagnosis: Upon the patient's presentation with dizziness and the discovery of a high‐grade atrioventricular block on the ECG, several potential conditions were considered, including arrhythmic events, transient ischemic attacks, and neurological disorders. The presence of cardiac conduction abnormalities prompted a focused differential that included brady‐arrhythmias and heart block, necessitating further cardiac‐specific diagnostic evaluations.

### 3.1 Investigations

– Electrocardiogram (ECG): An initial ECG confirmed a high‐grade atrioventricular block, guiding subsequent diagnostic and therapeutic interventions.
– Echocardiography: A bedside echocardiogram showed normal ventricular function and no valvular abnormalities, ruling out significant structural heart disease.
– Venography: Venography was performed during the pacemaker implantation to visualize the patient's venous anatomy, confirm the absence of the right SVC, and guide the lead placement strategy.
– Laboratory tests: Routine blood tests, including electrolytes and troponin levels, were conducted to exclude electrolyte imbalances and myocardial infarction. The results of these tests were within normal limits, further supporting the primary diagnosis related to the conduction system.

### 3.2 Treatment

The patient required a temporary pacemaker insertion due to an unstable and wide QRS escape rhythm. The temporary pacemaker was inserted via the right femoral vein to stabilize the patient's cardiac rhythm. The patient's venous anatomy presented complexity for the permanent pacemaker implantation, with an isolated PLSVC draining into the RA. As a result, a customized approach was necessary, involving the following:

– Access route: The left subclavian vein was used for vascular access, as the typical right‐sided approach was not feasible.
– Lead placement techniques: Specialized techniques were employed, including using an L‐shaped stylet to navigate the lead into the RV through the challenging anatomy of the PLSVC and coronary sinus.
– Choice of leads: An active fixation lead was chosen for secure placement in the RVOT to accommodate the anatomical challenges and ensure stable long‐term pacing.
– Fluoroscopic guidance: Extensive use of fluoroscopy was made to confirm the correct positioning of the leads and ensure optimal pacing parameters were achieved.

## 4 Discussion

This case report aimed to detail our approach to implanting a pacemaker in a patient with a rare and complex anomaly of the thoracic venous system, a first for our center.

The PLSVC is the most common anomaly of the venous drainage system, affecting 0.5%–2% of the population. While various anatomic variations of PLSVC have been documented, the least common and rare is the isolated PLSVC, which presented a unique challenge in this case [1].

The PLSVC, especially in its isolated form, is often accompanied by other congenital heart defects in approximately 46% of cases. Additionally, it can be associated with cardiac conduction abnormalities [1, 2].

To avoid unexpected complications in the catheterization lab, performing thorough echocardiography to diagnose PLSVC before pacemaker implantation is crucial. Echocardiographic signs of PLSVC include a dilated coronary sinus visible in the parasternal long axis (PLAX) and apical windows and abnormal flow patterns seen in the suprasternal view. These findings can be confirmed by injecting contrast through the left arm, filling the coronary sinus before the RA [3].

When a PLSVC is present, complications can occur during pacemaker implantation. These complications may include difficulty securing a stable lead position and maintaining consistent capture. The pacemaker lead must take a circuitous route through the PLSVC to reach the RV, often requiring longer lead lengths to accommodate the curved direction of the ventricular line [2, 4, 5].

We used the left subclavian vein approach instead of navigating the lead through the acute angle between the right brachiocephalic vein and the PLSVC to make lead manipulation easier [2, 4, 6]. The most challenging part of the procedure was guiding the lead into the RV through the narrow‐angle between the coronary sinus ostium and the tricuspid valve. We used an L‐shaped stylet to help the lead enter the RV to address this [2, 4, 5]. Another critical decision was whether to use an active or passive fixation lead and where to secure it. We chose an active fixation lead [6] and positioned it in the RVOT for better stability and sustainability [5]. In contrast, we used a passive fixation method to place the right atrial lead in the right atrial appendage via the PLSV.

The procedure required extended radiation exposure and a longer duration than standard pacemaker implantations utilizing the right SVC [7]. At the 1‐month follow‐up, we found that the atrial and ventricular pacing lead parameters remained stable.

## 5 Conclusion

When implanting pacemakers, it is crucial to be aware of potential cardiac venous anomalies, which may be linked to cardiac conduction issues. Implanting pacemakers in these anomalies can be challenging, especially when inserting and securing right ventricular (RV) leads. Achieving the best and long‐lasting outcomes in such cases demands a high level of expertise and effective teamwork.

## Author Contributions

**Shayan Shahi:** conceptualization, investigation, writing – original draft. **Soroush Nematollahi:** conceptualization, writing – original draft, writing – review and editing. **Ali Vasheghani‐Farahani:** conceptualization, project administration, supervision.

## Ethics Statement

The patient was provided written informed consent. All images and data would be anonymous and masked. The study was carried out according to the Declaration of Helsinki.

## Conflicts of Interest

The authors declare no conflicts of interest.

## Data Availability

The data of the present case are available upon reasonable request from the corresponding author.
